# Recent Advance on Metal Carbides Reinforced Laser Cladding Coatings

**DOI:** 10.3390/molecules30081820

**Published:** 2025-04-18

**Authors:** Dazhi Jiang, Guangjin Wang, Wei Dong, Xiaodong Hong, Chenguang Guo

**Affiliations:** 1 School of Mechanical Engineering, Liaoning Technical University, Fuxin 123000, China; 2 School of Materials and Energy, Foshan University, Foshan 528000, China; 3 School of Materials Science and Engineering, Liaoning Technical University, Fuxin 123000, China

**Keywords:** laser cladding coating, metal carbides, reinforcing agent, composite coating

## Abstract

The laser cladding technique can be adapted to fabricate composite coatings on the surface of the metal substrate, which not only effectively improves the surface properties of materials, but also greatly expands their application range. Metal carbides exhibit extremely high hardness, melting point, and outstanding chemical stability. The hardness of carbides is much higher than that of general metal materials. Therefore, various metal carbides serve as reinforcing agents for enhancing the overall performance of metal-based coatings. To date, there is no special review about metal carbide-reinforced laser cladding coatings. In view of the outstanding performance and wide application of metal carbides in laser cladding coatings, herein, recent advances in various metal carbide-reinforced metal coatings are highlighted. According to the type of metal carbides, the whole review is classified into five sections: WC-reinforced coatings, TiC-reinforced coatings, NbC-reinforced coatings, Ti_n+1_AlC_n_ (MAX) reinforced coatings, and Cr_3_C_2_, TaC-reinforced coatings. The preparation method, microstructure feature, and application performance of various carbide-reinforced composite coatings are summarized. At last, some prospects are put forward on the current issues and future development directions, aiming to provide comprehensive and in-depth references for the research and application in the field of composite coatings.

## 1. Introduction

With rapid technological development, the requirements for material performance in various industrial fields are becoming increasingly stringent. Traditional materials often struggle to meet the high-performance requirements of wear resistance, corrosion resistance, and high-temperature resistance under complex working conditions. Therefore, material surface modification technology has emerged as a key strategy to improve the comprehensive performance of materials. Laser cladding, as an advanced surface modification technology, has received much attention in recent years. It can accurately prepare coatings with specific properties on low-cost substrate materials, which not only effectively improves the surface properties of materials, but also greatly expands their application range [1]. When a high-energy laser beam is irradiated onto the surface of the substrate material, a huge amount of energy is instantly released, causing a very small area of the substrate surface to rapidly heat up to a molten state. At the same time, the alloy powder is transported to the laser action area through a powder feeding device, and the alloy powder quickly melts at high temperatures and is fully mixed with the melted matrix material. With the movement of the laser beam, the molten pool rapidly cools and solidifies, forming a layer of fusion coating on the surface of the substrate that is firmly metallurgically bonded to the substrate. This metallurgical bonding method ensures good bonding strength between the cladding layer and the substrate, effectively transmitting stress and jointly bearing external loads [2]. With the continuous advancement of laser technology, from the initial simple preparation of single component cladding layers to the development of complex multi-element alloy systems and gradient functional coatings today, laser cladding technology has become increasingly important in the field of material surface modification, continuously driving the technological upgrading and development of related industries.

The performance of laser cladding coatings is closely related to their composition, which is complex and diverse. Different components work together to determine the various characteristics of the coating [3]. Laser cladding coatings are mainly composed of substrate, cladding material, and additives. The substrate material is the fundamental support for laser cladding coatings, which not only provides physical support for the coating, but also has a significant impact on the bonding strength between the coating and the substrate. Usually, the substrate material is the workpiece itself that needs surface strengthening or modification, commonly including various metal materials such as steel, aluminum alloy, titanium alloy, etc. Cladding materials are the core part of laser cladding coatings, and they come in various types. Different types of cladding materials can be selected according to different usage requirements, such as metal powder, ceramic powder, and composite material powder. During a laser cladding process, special additives, such as deoxidizer, alloying element, and activator, are often added to improve the quality and performance of the coating [4].

Ceramic materials have the advantages of high hardness, high melting point, high-temperature resistance, wear resistance, corrosion resistance, etc. Therefore, ceramic powders have also been widely served as reinforcing agents for preparing high-performance composite coatings. Commonly used ceramic powders include aluminum oxide (Al_2_O_3_), zirconium oxide (ZrO_2_), tungsten carbide (WC), etc. Among the existing ceramic powders, metal carbides exhibit extremely high hardness, high melting point, and good chemical stability. The hardness of some carbides such as WC and TiC is much higher than that of general metal materials. When these carbides are added to the laser cladding coating, the overall hardness of the coating is significantly improved [5]. In addition, in the actual process of friction and wear, hard carbide particles act as a hard shield to resist the cutting and plowing effects of abrasive particles. Even under harsh wear conditions, carbide particles still maintain relative stability, effectively protecting the coating substrate and greatly reducing the wear rate of the coating. This makes metal carbide-enhanced laser cladding coatings have significant advantages in applications that require high wear resistance, such as mining machinery, mold manufacturing, etc. In fact, metal carbides in laser cladding coatings play three functions: diffusion strengthening effect, refining grain effect, and improving interface integration. Thus, metal carbides are widely used to enhance the performance of laser cladding coatings, and metal carbide-enhanced laser cladding coatings have become one of the research hotspots in the field of materials science.

**Figure 1:**
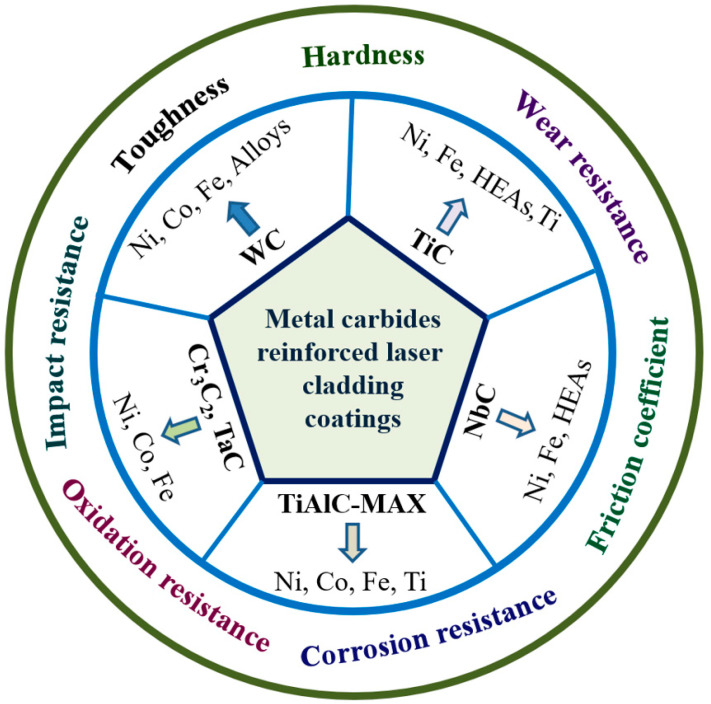
The scheme of various metal carbide-reinforced laser cladding coatings.

To date, there is no special review about metal carbide-reinforced metal composite coatings. In view of the outstanding performance and wide application of metal carbides in laser cladding coatings, herein, the research progress of various metal carbide-reinforced composite coatings is highlighted. According to the type of commonly used metal carbides, five sections are divided, including WC-reinforced coatings, TiC-reinforced coatings, NbC-reinforced coatings, Ti_n+1_AlC_n_ (MAX)-reinforced coatings, and Cr_3_C_2_- and TaC-reinforced coatings, as shown in Figure 1. The preparation method, microstructure feature, and application performance of various carbide-reinforced metal composite coatings are summarized in detail.

## 2. WC-Reinforced Composite Coatings

The Mohs hardness of WC is between 8.5 and 9.5, second only to diamond, which makes it perform well in various wear environments. Its high hardness enables WC-containing coatings to effectively resist abrasive wear, adhesive wear, and fatigue wear. Furthermore, the coatings containing WC exhibit superior corrosion resistance, high-temperature resistance, good bonding strength, and surface smoothness [6]. Thus, WC-reinforced composite coatings have been widely used in fields such as mechanical components, cutting tools, and molds.

### 2.1. WC/Ni Composite Coatings

As an important means of material surface protection and strengthening, Ni-based coatings have numerous significant advantages. Excellent corrosion resistance is the most prominent characteristic of Ni-based coatings. Ni itself has good chemical stability. In different corrosive environments, Ni-based coatings can form dense and stable passivation films, effectively preventing external corrosive media from contacting the substrate material and greatly extending the service life of the substrate material. Ni-based coatings also have excellent high-temperature resistance [7]. Under high-temperature conditions, Ni-based coatings can maintain good mechanical properties and chemical stability. In high-temperature working environments, Ni-based coatings not only protect the substrate material from oxidation but also maintain the structural integrity and mechanical properties of components, ensuring the normal operation of equipment. Good wear resistance is also a major advantage of Ni-based coatings. By designing the coating composition and microstructure reasonably, Ni-based coatings can have high hardness and toughness. During the process of friction and wear, Ni-based coatings can effectively resist wear, reduce the wear rate of material surfaces, and improve the wear resistance life of components. In addition, Ni-based coatings usually have good adhesion with the substrate material, which can firmly adhere to the surface of the substrate and is not easy to peel off. This ensures the reliability and stability of the coating during long-term service. Ni-based coatings play an indispensable role in modern industry due to their many advantages [8]. All kinds of Ni/WC composite coatings have been reported to boost the mechanical performance of metal materials. To develop high-performance Ni/WC composite coatings, some basic issues have been explored, including the influence of WC content and particle size, the optimization of process parameters, the addition of WC and other ceramic phases, and so on.

**Figure 2:**
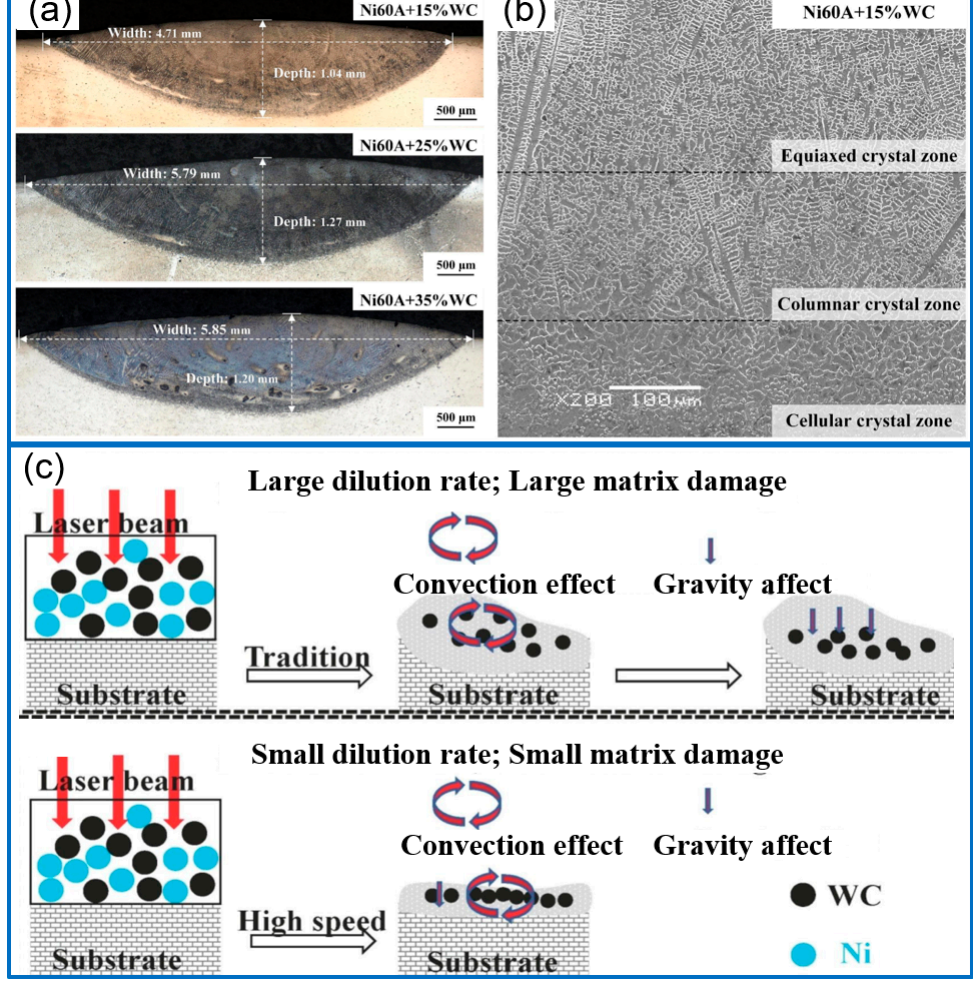
Effect of WC content on the composite coatings (**a**) and microstructure distribution (**b**) [9]; Copyright (2024) Elsevier. Convection and gravity model of Ni/WC composite coating during solidification, traditional laser cladding and HSLC (**c**) [10]; Copyright (2022) Elsevier.

To reveal the influence of WC content, Wang et al. [9] prepared Ni60A/WC coatings on the substrate of Inconel 718 alloy and revealed the influence of WC content on the WC/matrix micro-interface evolution, microhardness, and corrosion resistance. The microstructures of different samples are provided in Figure 2a,b. The result indicated that the coating with 35% WC presented the highest microhardness of 375 HV0.5. While the coating of Ni60A + 15% WC exhibited the best corrosion resistance. Hu et al. [10] fabricate Ni/WC composite coatings on 304 steel through a high-speed laser cladding method, as described in Figure 2c. During the thermal processing, WC particles decomposed and generated the WC, W_2_C, and Cr_23_C_6_ strengthening phases. The increasing WC fraction would refine the coating structure. When the WC content was less than 30%, the wear resistance and hardness of the coating were gradually improved with an increasing WC content. Liu et al. [11] fabricated Ni50-WC coatings and investigated the influence of WC content on morphology, corrosion, and mechanical performance. Through comparison, the coating of Ni50-20WC showed a higher microhardness (817.9 HV) and a lower friction coefficient than that of Ni50-10WC coating, due to the generation of fine and compact structure. Furthermore, the Ni50-20WC coating presented an outstanding corrosion resistance. Li et al. [12] prepared Ni45/WC coatings on the substrate of 42CrMoA to enhance the impact resistance and wear resistance. When the WC content was 12 wt%, the coating exhibited a lower wear rate (91.59%) than that of the Ni45 coating. However, the coating containing 12 wt% WC presented a poor impact resistance. Regarding the performance of the coating with a high content of WC, Shen et al. [13] fabricated three NiCrSiBC/WC composite coatings on the carbon steel with a WC ratio of 40%, 50%, and 60%. When the content of WC was enhanced to 50% from 40%, the tensile stress of coating increased and the fracture toughness significantly reduced, causing an increase in crack susceptibility. When it reached 50%, coating hardness would not increase with an increase in WC fraction, while the cracking susceptibility increased, which was attributed to more serious cracks generated.

The influence of WC particle size on the performance of Ni/WC coating on 304 steel was disclosed by Tan [14]. Three kinds of particle sizes were selected as fillers, including 100–145 μm, 65–100 μm, and 30–65 μm. The content of WC was fixed as 40% and the matrix was Ni1725 powder. Through comparison, the Ni/WC coating made by the size of 30–65 μm exhibited the highest microhardness, the lowest friction coefficient, and wear volume, among the three coatings fabricated by different WC particles.

Except for the study on the WC content and particle size, the structure design of WC-reinforced coating is considered a crucial factor for determining the performance. In this respect, Wang et al. [15] fabricated Ni-WC gradient composite coating on the substrate of Q345R steel. The first layer was C276/10WC, and the subsequent layers were Ni60/10WC, Ni60/30WC, and Ni60/50WC. Gradient composition coating effectively reduced the cracking tendency. The microhardness of the resulting gradient coating reached 1053.5 HV_0.2_, about four times that of the Q345R. Furthermore, the wear loss was 82.7% of the Q345R.

In addition to the influence of coating composition, the effect of process parameters on the performance was also investigated. For example, Bian et al. [16] fabricated Ni60-WC composite coatings on H13 steel and discussed the influence of powder feeding rate on the microhardness of coating. The result demonstrated that the feeding rate affected the generation of new compounds in composite coating. When operated at 25 g/min, more compounds were produced, and the resulting coating presented the maximum microhardness of 936HV, which was 2.4 times that of the substrate H13.

To overcome the shortage of single WC, other ceramic phases or rare-earth activators have been introduced to further enhance the mechanical performance of laser cladding coatings. Li et al. [17] designed Ni62/WC+ TiC composite coatings by cladding on 65Mn steel. The ratio of WC/TiC was fixed as 3:1, and the mass fraction of the reinforcing phase was 10%, 20%, and 30%. The coating containing 20 wt% reinforcing phases had the highest microhardness of 1000 HV1, three times higher than that of 65Mn steel. Moreover, this coating also presented the lowest wear rate. In addition, Cr_3_C_2_ and WC acted as reinforcing phases for fabricating Ni/WC + Cr_3_C_2_ coating on the surface of Q235 steel [18]. When 5% Cr_3_C_2_ was introduced into Ni/WC, the plastic deformation of the Ni-3 coating was prohibited. Furthermore, this coating exhibited the lowest weight loss and the highest microhardness of 831HV_0.1_. In Chen’s work [19], in situ-generated NbC enhanced Ni-WC composite coating was prepared on AISI 1045 steel, under the presence of Nb, C, WC, and Ni50A powder. Through adjusting the ratio of Nb and C, different amounts of NbC were in situ-synthesized in composite coating. The result confirmed that the optimum Nb/C ratio was 1: 2, and the resulting coating possessed the best wear resistance and hardness. They also fabricated (Nb, M)C (M = Ti, V, and Zr) reinforced Ni-WC coatings by introducing other metal carbides [20]. A core-shell structure was formed in (Nb_0.5_Ti_0.5_)C coating with W_p_C-shell and (Nb_0.5_Ti_0.5_)C-core, and this coating possessed the best wear resistance and hardness. In addition to the reinforcing agents, rare-earth La_2_O_3_ can be used to improve the corrosion resistance and wear of the WC-containing coatings. In this respect, Zhao et al. [21] prepared La_2_O_3_-doped Ni60/WC coatings on the substrate of AISI 304 steel. Compared with the coating of Ni60/WC+ nano-La_2_O_3_, the micron-La_2_O coating had a higher microhardness, better corrosion resistance, and wear resistance, due to the fine grain strengthening effect.

### 2.2. WC/Co, WC/Fe Composite Coatings

**Figure 3:**
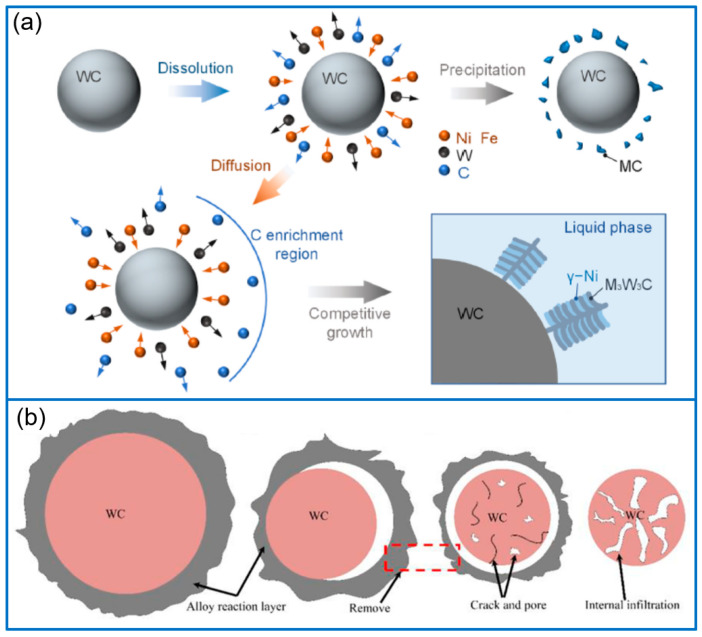
Scheme of carbide formation mechanism (**a**) [24]; Copyright (2021) Elsevier. The slight reduction in the size of WC particles, and defects such as ablation cracks and pores appear on WC particles (**b**) [26]. Copyright (2021) Elsevier.

In addition to the WC/Ni-based composite coatings, WC-reinforced Co-based and Fe-based coatings have been widely reported in recent years. For example, Jiang et al. [22] prepared the WC-Co composite coatings on Ti-6Al-4 V alloy substrate by laser cladding. In their work, WC particles were uniformly wrapped by the Co phase and denoted as WC-17Co, with a diameter of 80 μm. Thanks to the synergistic role of fine microstructure and metallurgical bonding, the microhardness of coating material reached 1536 HV_0.5_, and the wear rate was as low as 1.5 g/h. Lv et al. [23] fabricated WC/Co-based coatings on 42CrMo steel by using the laser cladding technique, and discussed the influence of direct addition and in situ synthesizing WC on the coating performance. Through comparison, the optimal coating made by the direct addition method exhibited a higher microhardness and better wear resistance. The wear resistance and microhardness of optimal coating were 3.75 times and 1.46 times higher than that of 42CrMo steel, respectively. In another work, various WC-12Co coatings were prepared on the Inconel 718 substrate [24], and the WC-12Co fraction ranged from 0 to 30 wt%. The introduction of WC-12Co would refine the microstructure and prevent the growth of columnar grains, as shown in Figure 3a, further improving the hardness and wear resistance. Through comparison, the coating containing 30%WC-12Co had the highest microhardness of 462.63 HV_0.5_ and the best wear resistance. In addition to the simple composite coating of Co/WC, Ding et al. [25] developed a Co-based alloy/WC/CaF_2_ composite coating with a mass ratio of 85/10/5. During the laser cladding process, the F element was dissolved in the α-Co phase and the coating was composed of Cr_23_C_6_, α-Co, and WC phases. The composite coating had a high microhardness of 600 HV0.2. Furthermore, the resulting composite coating greatly enhanced the fretting wear resistance at both room temperature and high temperature of 500 °C.

Compared with Ni-based and Co-based alloy powders, Fe-based alloy powders have the advantages of high performance and low cost. There are lots of reports about WC-reinforced Fe-based coatings. In this field, Xiao et al. [26] fabricated Fe-WC coatings on 15CrNiMo steel via laser cladding and discussed the influence of WC fraction on the microhardness, structure, and wear resistance of the composite coating. The addition of WC greatly enhanced the microhardness of Fe-based coating, even increased to 1029.2 HV_0.2_ from 729.9 HV_0.2_. Furthermore, the wear resistance of WC-Fe composite coating was 1.3 times that of Fe coating. The transition mechanism of WC particles is presented in Figure 3b. The enhancement of wear resistance was ascribed to the presence of WC, W_2_C, M_7_C_3_, M_23_C_6_, and η phases. By laser cladding the mixed powders of Fe60 and WC, Li et al. [27] in situ synthesized Fe-(Cr, W)_23_C_6_-WC composite coatings on the substrate of 16Mn steel. When the WC content was 20 wt%, the composite coating had the maximum hardness and the best wear resistance. The improved performance was attributed to the in situ generation of network-shaped (Cr, W)_23_C_6_-WC ceramic phases in α-Fe dendritic matrix, and the evolution process of phase was WC _primary_ + liquid_0_→WC _in situ_ + liquid_1_→WC _in situ_ + liquid_2_ + γ-Fe→WC _in situ_ + M_23_C_6_ + α-Fe. Based on the Fe/TiC composite coating, Zeng et al. [28] prepared Fe50/TiC/WC ternary coating on the substrate of AISI 1045 steel and investigated the influence of the WC fraction on microhardness and the friction coefficient. Through optimization, the Fe50/TiC/WC ternary coating containing 10% WC presented the best performance. Compared with the pristine substrate, the microhardness was enhanced three times, the friction coefficient was 27%, and the wear volume was only 1.5%.

### 2.3. WC/Other Metal Composite Coatings

H13 is a commonly used tool steel with excellent wear resistance and high-temperature strength. Based on the H13 powder matrix, WC and Y_2_O_3_ powder were selected as reinforcing phases, and H13/WC + Y_2_O_3_ composite coating was reported to modify 8407 steel [29]. The presence of Y_2_O_3_ promoted the decomposition of WC, leading to the formation of uniform coating. By adjusting the content of Y_2_O_3_ and WC, the coating made by H13 + 1%Y_2_O_3_ + 10%WC exhibited the best performance. The hardness at room temperature and 500 °C was 48.4% and 32.3% higher than that of the 8407 steel, respectively. The improved hardness was ascribed to the generation of the M_7_C_3_ strengthening phase.

Eutectic high-entropy alloys (EHEAs) have been considered a new type of alloy with a promising application potential, due to the combination properties of high-entropy alloys and eutectic alloys [30]. To further enhance the mechanical performance of EHEAs, WC-reinforced coating has been fabricated on their surface via laser cladding technique. For example, Li et al. [31] developed AlCoCrFeNi_2.1_/WC EHEAs composite coatings for modifying the 316 L stainless steel. With an increasing WC content, the Cr_21_W_2_C_6_ and Cr_7_C_3_ phases were gradually generated in the composite coating. The composite coating containing 30 wt% WC possessed a maximum hardness of 572.3 HV_1.0_ and the best wear resistance.

Invar alloy, also known as low expansion alloy, is a magnetic metal alloy composed of iron (Fe) and nickel (Ni). Its main components are 36% nickel and 64% iron, with a face-centered cubic structure. The outstanding feature of Invar alloy is its extremely low coefficient of thermal expansion, which makes it suitable for applications that require extremely high precision. Based on the matrix of Invar alloy powders, WC and Y_2_O_3_ were used as reinforcing agents for improving the surface properties of the Invar alloy substrate [32]. When Y_2_O_3_ powder is added, the coating thickness and melting efficiency can be improved. Moreover, a high fraction of Y_2_O_3_ would induce the transformation from cellular dendritic crystals to cellular crystals. When the Y_2_O_3_ fraction was 5%, the microhardness of the composite coating was 193.1 HV, which was much higher than that of the substrate.

To sum up, all kinds of WC-reinforced metal composite coatings have been introduced in this section. According to the types of metal matrix, several parts are classified, including WC/Ni, WC/Co, WC/Fe, and WC/other metal composite coatings. Compared with another metal matrix, many more works focus on the development of WC-reinforced Ni composite coatings. Furthermore, various alloy powders, such as H13, HEAs, and Invar alloy have been selected as metal matrices for fabricating WC-reinforced alloy coatings. Among this literature, the research direction mostly involves the effect of WC content and particle size, the optimization of process parameters, coating structure design, a combination of WC and other ceramic phases or oxides, in situ synthesis of WC, and the selection of metal matrix, and so on. Regarding the performance enhancement of WC-reinforced composite coatings, most works have discussed the influence of WC addition on hardness, tensile stress and fracture toughness, wear resistance, corrosion resistance, and so on. Considering the difference in testing conditions and metal matrix, it is very difficult to compare the coating performance in different literature.

## 3. TiC-Reinforced Composite Coatings

TiC has the merits of high hardness, high strength, high melting point, corrosion resistance, and wear resistance, which is widely used in the mechanical industry, aerospace, building materials, and electrical fields. TiC-reinforced metal composite coatings exhibit excellent hardness and wear resistance, good high-temperature resistance, outstanding chemical stability, and low friction coefficient. The preparation methods of TiC-reinforced coatings can be divided into direct feeding method and in situ synthesis method. In this section, the recent advances in various TiC-reinforced laser cladding coatings are introduced, including Ni-based, Fe-based, high-entropy alloys based, and Ti-based coatings.

### 3.1. TiC/Ni Composite Coatings

Among the existing works about TiC/Ni composite coatings, based on the feeding method, direct feeding method, and in situ generation method can be classified. Furthermore, the research topics mostly focus on the TiC content and particle size, process parameters, in situ reinforcement of TiC, and so on. Regarding the direct addition of TiC, Cai et al. [33] discussed the influence of TiC fraction on the structure and performance of TiC/Ni60 composite coating. Various precipitates were generated in the coatings with the change in TiC content. As the TiC content increased, the wear resistance and bending strength both decreased. The main reason was attributed to the stress concentration around the large-size particles, and the generated cracks were easily extended, leading to brittle fractures. Meng et al. [34] investigated the effect of the nano-TiC addition method on the performance of Ni-based self-fusing alloy coatings. Through comparison, the Ni4 sample with chemical electroless coating of Ni-B-TiC presented the optimal wear resistance and the maximum microhardness, due to the bimodal structure generated by the nonuniform dispersion of nano TiC in the composite coating. To obtain the optimum composite coating, Chen et al. [35] discussed the influence of process parameters on the performance of TiC/Ni45 composite coating. In their work, 10 wt% TiC and Ni45 self-fluxing powder were cladding on the surface of 45 steel. The best parameters were obtained: powder feed rate 243.7 mg/s, laser powder 380 W, and scanning speed 5 mm/s. The microhardness of the resulting coating was about 2.5 times that of 45 steel.

**Figure 4:**
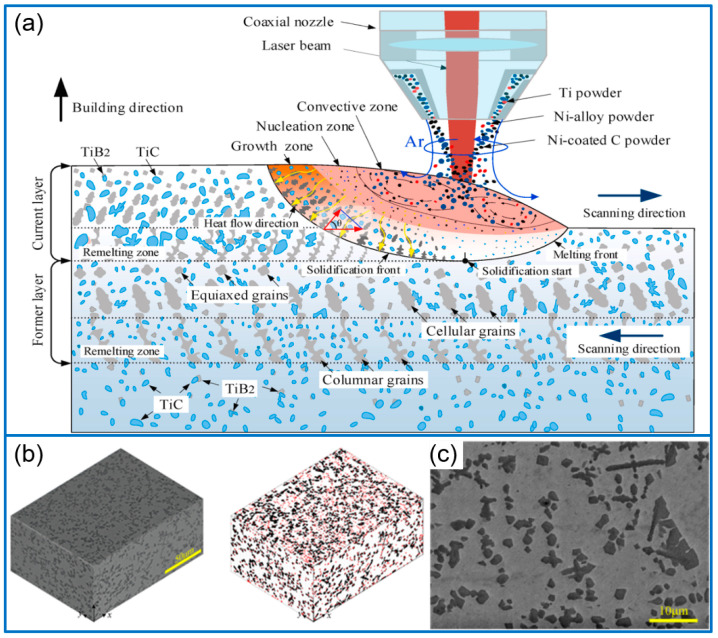
The effect of in situ TiC on the microstructure of the remelting zone between the layers of TiC/Ni coating (**a**) [36]; Copyright (2022) Elsevier. Microstructure of in situ TiC-reinforced coating (**b**,**c**) [37]. Copyright (2025) Elsevier.

Except for adding TiC powders directly, TiC can be in situ synthesized in the Ni matrix. In this field, Chen et al. [36] fabricated TiC/Ni functionally gradient coatings (FGC) by using Ni45 powder, Ti particles, and Ni-coated graphite, and the coating microstructure of the remelting zone is given in Figure 4a. The microhardness of the TiC/Ni coating gradually increased from the bottom to the top, and the maximum value reached 1036.25 HV. The wear rate gradually decreased from the bottom to the top, and the top of the coating exhibited the smallest wear rate of 4.872 × 10^−6^ mm^3^·N^−1^⋅m^−1^. To prepare TiC/Ni composite coating, Ti powder, and Ni-coated graphite were mixed with In625 matrix to in situ synthesize the TiC phase. The microstructure is shown in Figure 4b,c. The robust bonding between matrix and in situ-generated TiC would remove the plastic of conventional coatings by grinding method [37]. In addition, Wang et al. [38] fabricated in situ TiC-reinforced coating by using the mixed powders of 96 wt% TC4 and 4 wt% SiC. During the thermoforming process, SiC was decomposed to produce TiC in situ. The optimized C16 coating obtained at 1600 W exhibited the highest microhardness (351.8 HV_0.2_), with a 38.1% wear rate of the substrate.

To further enhance the mechanical property of TiC-reinforced coating, other ceramic phases can be in situ introduced into the metal matrix. For example, various fractions of TiB_2_-TiC were in situ synthesized in the Ni-based alloy matrix by adjusting the dosage of Ni-coated graphite, Ti, and B_4_C [39]. The in situ TiB_2_-TiC coating possessed an enhanced corrosion resistance. Moreover, the wear volume was 46.4% lower than that of pure Ni-based coating. Qi et al. [40] also fabricated TiC-TiB_2_ binary phases reinforced Ni-based coating by using Ni-coated graphite, TA0, and B_4_C powders. A high Ni-coated graphite fraction would decrease the nucleation rate and the size of the ceramic phase. Through comparison, the reinforced coating prepared by 8 wt% Ni-coated graphite and 3 wt% B_4_C presented the best wear resistance, and the wear volume had a 26.47% reduction. Moreover, the corrosion potential increased by 39%, and corrosion current density decreased significantly. Zheng et al. [41] synthesized TiB_2_- and TiC-reinforced Ni50A composite coating on AISI 1045 steel by using the powders of Ni50A, Ti, and B4C. The influence of scanning rate, laser power, and Ti/B_4_C powder ratio on the coating properties was investigated. By comparing the hardness and corrosion resistance of different coatings, the best process parameters were obtained. The Ti/B_4_C ratio was 4:1, the scanning rate was 8 mm/s, and the laser power was 1200 W.

In addition to the Ni powers, various Ni-based alloy powders have been selected as matrices to prepare TiC/Ni-based alloy composite coating. For example, to improve the erosion resistance and hardness of AISI 420 stainless steel, NiCr-TiC composite coating was prepared by laser cladding of NiCr-TiC powders on a stainless steel surface [42]. The result demonstrated that the hardness of the coating significantly increased with the addition of TiC particles. During the experiment in the erosion test, the NiCr-TiC coating presented the lowest mass loss, while pure AISI 420 stainless steel had the maximum mass loss. In addition, TiC/Inconel 625 composite coating was fabricated by extremely high-speed laser cladding technology (EHLA) toto improve the corrosion resistance of AISI 1045 [43]. The high-speed cladding accelerated the solidification rate of the coating and the generation of carbides. The generated CrC and NbC grains effectively blocked the corrosion channels and improved anti-corrosion performance. Compared with the coating prepared by conventional laser cladding, the EHLA coating exhibited better corrosion resistance.

### 3.2. TiC/Fe Composite Coatings

TiC can be used for enhancing the mechanical performance of Fe or stainless steel matrix. In this respect, Zhu et al. [44] discussed the influence of TiC content on the reinforced 410 martensitic stainless steel (MSS) coatings. When the TiC content was up to 15%, pores and microcracks would be formed in the coating. The increasing TC content in the coating would enhance the wear resistance and microhardness. Through comparison, the coating containing 10% TiC possessed the best comprehensive performance, much better than that of pure MSS coating. Hui et al. [45] fabricated WC-10Co_4_Cr-xTiC coatings on the substrate of H13 steel by adding TiC into WC/10Co_4_Cr powders. The relationship between the wear resistance and TiC fraction was disclosed. The result showed that the porosity of the coating was reduced after adding TiC, and the composition of the coating was W_2_C, WC, Cr_7_C_3_, Co_3_W_3_C, and TiC. The friction coefficients increased and the wear rate decreased.

Based on the matrix of ferrite stainless steel (SMC) powder, Ti powder and Cr_3_C_2_ powder were introduced to in situ synthesize TiC-reinforced SMC coatings [46]. The generation of TiC was related to the replacement of Cr in the reaction process, which led to the improvement of the corrosion resistance of the coating. Furthermore, with an increase in TiC content, the wear resistance and hardness of composite coating were greatly improved. In addition to generating a single TiC-reinforcing phase, Yang et al. [47] fabricated TiC and VC binary phases reinforced Fe-Cr-C alloy (2Cr13), in which, nickel-coated graphite, Ti, and V powders (C/Ti/V molar ratio of 2:1:1) were used to in situ generate composite coating with a high wear resistance. The optimized composite coating containing the 5% group exhibited better wear-resisting properties. The abrasion resistance was even enhanced to more than 21 times higher than that of semi-steel roll material (160CrNiMo) substrate. Jiang et al. [48] prepared TiC/NbC coating on 304 stainless steel by using the 304 powder, B_4_C powder, TC4 powder, and Nb powder. The hard phases Cr_7_C_3_ and Cr_23_C_6_ were produced with an increasing (Ti, Nb) C-reinforcing agent, which facilitated the enhancement of hardness. In addition, the friction coefficient and wear rate of the coating were affected by the laser power. In another work [49], B4C, Ti powder, and 316 L stainless steel powder were used to in situ synthesize TiC/TiB_2_-reinforced composite coatings, and the resulting composite coating contained the phase of TiB_2_, TiC, Fe_2_B, Cr_3_C_2_, and Fe_3_C. The 2# coating prepared by adding 5%B_4_C-15%Ti composite powder exhibited the highest microhardness of 961.94 HV_0.3_, and the sample also presented the smallest volume loss and smallest friction coefficient among all samples.

### 3.3. TiC/High Entropy Alloys Composite Coatings

**Figure 5:**
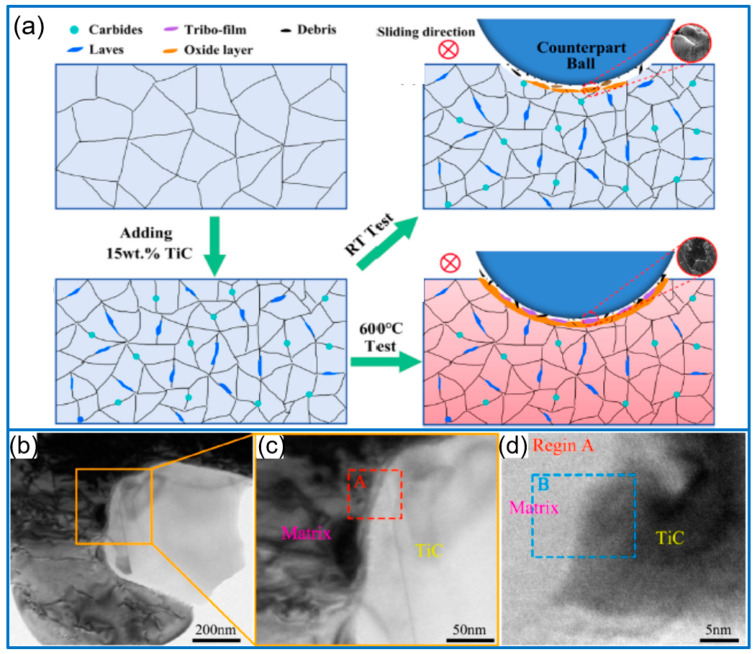
Microstructure evolution for TiC-free coating and 15 wt% TiC coating, and the wear behaviors at different temperatures (**a**) [50]; Copyright (2024) Elsevier. TEM images of TiC and HEA matrix (**b**), and corresponding magnified region (**c**,**d**) [52]. Copyright (2024) Elsevier.

TiC-reinforced high-entropy alloys (HEAs) coatings have been well focused in recent years. In this field, Chen et al. [50] developed TiC-reinforced FeCoCrNiCu HEA composite coatings by adjusting the fraction of TiC particles, and revealed their wear resistances at RT and 600 °C, respectively, as shown in Figure 5a. The coating with 15 wt% TiC exhibited a superior wear resistance at RT, and the microhardness was 531.65 HV_0.5_. When tested at 600 °C, TiO_2_ and the Cr_2_O_3_ tribo-film generated on the coating surface effectively resisted the friction by facilitating the lubrication. In addition to directly adding TiC particles, TiC particle-reinforced AlCoCrFeNi-based coatings were in situ synthesized on the substrate of 40CrNiMo by using the Ti powder and Cr_3_C_2_ [51]. The in situ-generated TiC particles greatly improved the wear resistance and hardness. In addition, Gao et al. [52] fabricated in situ TiC-reinforced CoCrFeNi HEA composite coatings by using Ti powder, C powder, and CoCrFeNi HEA powder. During the laser cladding process, the Cr_7_C_3_ and TiC phases were formed in the coating. Different from directly adding TiC particles, in situ-generated TiC promoted the nucleation rate and facilitated the refinement of grain size (Figure 5b,d). The enhanced microhardness and wear resistance were ascribed to the fine grain, second phase strengthening, and solid solution strengthening. Li et al. [53] prepared WC/TiC composite reinforced AlCoCrFeNi coating by adding Ti powder and WC, and investigated the microstructure, wear resistance, and hardness of the resulting coating. Due to the presence of WC and in situ-generated TiC particles and solid solution strengthening, the hardness and wear resistance of AlCoCrFeNi-WC/TiC coating enhanced to 808.7 HV_0.3_ and 14 times, respectively. In addition, W and in situ TiC-reinforced CoCrFeNi HEA coatings were synthesized on 45 steel [54]. The quality of composite coating and its hardness and wear resistance were significantly improved by adding W and in situ TiC, due to the synergistic effect between TiC and W. The highest hardness of the coating reached 1122.43 HV_0.2_.

Except for the change in TiC fraction and the type of HEAs, Zhuang et al. [55] investigated the influence of ultrasound on TiC distribution in 4 wt% TiC-reinforced CrMnFeCoNi coatings. Ultrasonic amplitude greatly affected the distribution of TiC particles. A low amplitude of 5 μm would lead to the particle aggregation. When enhanced to 10–20 μm, the fishbone-shape distribution of TiC was obtained. In addition, the increased ultrasonic amplitude would increase the hardness and reduce the corrosion resistance of the composite coating.

### 3.4. TiC/Ti Alloys Composite Coatings

Among the existing metal materials, Ti and Ti alloys can serve as biomedical materials and structural materials for chemical engineering, shipbuilding, aerospace, etc. Among Ti alloys, TC4 (Ti-6Al-4V) exhibits superior corrosion resistance, heat resistance, and strength. However, the poor wear resistance and low hardness further limit their wide application. Considering the good wettability of Ti alloys and TiC-reinforcing agents, Chen et al. [56] fabricated TiC/TiAl coatings on TC4 substrate and revealed the influence of TiC fraction on the microstructure and performance of coating. Four phases of TiAl, Ti_3_Al, TiC, and Ti_2_AlC were formed in the coating. Through comparison, the coating containing 5 vol% TiC presented the lowest friction coefficient and wear rate. While the 15 vol% coating had the highest hardness of 799 HV_1.0_.

By using the Ti6Al4V alloy as the matrix, TiC/TiB composite biocompatible coatings were in situ synthesized with pure B_4_C and pure Ti powder as reactants [57]. In fact, the coating was composed of the TiB, TiC, Ti_3_Al, TiVC_2_, and α-Ti phases. The introduction of TiB greatly improved the mechanical performance of the coating, including microhardness and fatigue wear resistance. In addition, Zhang et al. [58] prepared TiC_x_-reinforced CrTi_4_-based composite coatings on Ti6Al4V substrate by using the powders of NiCr-Cr_3_C_2_, CeO_2_, and Ti6Al4V, and discussed the effect of CeO_2_ fraction on the microstructure of the coating. The addition of CeO_2_ did not affect the phase composition (TiC_x_ and CrTi_4_), while effectively preventing the generation of pores and cracks. The decomposed CeO_2_ would recrystallize to produce Ce oxides dispersed at the TiC_x_/CrTi_4_ boundary. Compared with the substrate of Ti6Al4V, the wear resistance and microhardness of TiC_x_-reinforced CrTi_4_-based coating were increased by 57% and 23% [59].

In summary, various TiC-reinforced metal composite coatings are summarized in this section, such as TiC/Ni, TiC/Fe, TiC/HEAs, and TiC/Ti alloy composite coatings. Unlike the WC-reinforced metal coatings, most of the reports have focused on the in situ synthesis of TiC by adopting different reactants, such as Ti powers, nickel-coated graphite, B_4_C, Cr_3_C_2_ or WC, and so on. Compared with the coating prepared by directly adding TiC, in situ-generated TiC facilitated the nucleation rate and achieved the refinement of grain size. Moreover, solid solution strengthening and second-phase strengthening also contributed to the enhancement of hardness and wear resistance. Therefore, TiC has been widely adopted as the reinforcing agent for enhancing the hardness and wear resistance of laser cladding coatings. Considering the advantages of in situ-generated TiC, in addition to the TiC-reinforced phases, other reinforced agents are introduced into the metal matrix, such as WC, Cr_3_C_2_, TiB_2_, NbC, VC, etc. Different from the WC, HEAs, and Ti alloys widely serve as the matrix for TiC, and lots of literature has reported the TiC/HEAs and TiC/Ti alloys composite coatings, further indicating the specialization and universality of TiC-reinforcing agents.

## 4. NbC-Reinforced Composite Coatings

As an important reinforcing agent, niobium carbide (NbC) has received widespread attention in the field of materials science, due to the advantages of high melting point (3490 °C), high hardness (24.6 GPa), and outstanding mechanical properties. Adding NbC-reinforcing agent to metal materials can form small and uniformly distributed NbC particles. These particles effectively hinder dislocation motion, thereby significantly improving the strength and hardness of metal materials. To date, various NbC-reinforced metal coatings have been developed, including NbC/Ni composite coatings, NbC/Fe composite coatings, and NbC/HEAs composite coatings.

### 4.1. NbC/Ni Composite Coatings

NbC acts as a reinforcing agent for improving the mechanical performance of Ni-based coating by direct addition or in situ synthesis method. For example, Gaddam et al. [60] fabricated NbC/Ni composite coating through the in situ reaction between 90 wt% Ni and 10 wt% NbC. The authors disclosed the microstructure and the phase evolution of composite coating. By using Ni-coated graphite, Ni625, and Nb powder as raw materials, NbC-reinforced Ni-based coating was in situ synthesized on the 42CrMo steel [61]. The amount of additive greatly affected the corrosion resistance and mechanical performance of the coating. When the NbC amount was 15%, the resulting coating had the highest hardness of 441.9 HV. While the best corrosion resistance was obtained with the addition of 10%. When the addition was 20%, few NbC would be formed in the coating, which led to the degradation of wear resistance and hardness of the coating. In addition, Lian et al. [62] also prepared NbC-enhanced Ni-based coatings by using B_4_C and Nb powders and discussed the influence of Nb and B_4_C dosage on the coating performance. The hardness could be improved by increasing the content of B_4_C, and the highest hardness reached 1794.1 HV_1.0_. Different from the change of hardness, wear resistance would increase firstly with an increasing B4C content, and then reduce. when the Nb/B4C ratio was 1:1.3, the resulting coating presented the best wear resistance. The authors also focused on the research on in situ xNbC-reinforced Ni-20WC coating [63], and revealed the influence of NbC content on the structure, hardness, and corrosion resistance of the composite coating. An increased NbC fraction would lead to the agglomeration of NbC particles, surface roughening, and WCp nucleation. When x was 20 wt%, a refined structure was obtained with uniform distribution. The resulting coating had an enhanced microhardness (3.6 times of matrix) and optimal corrosion resistance.

### 4.2. NbC/Fe Composite Coatings

**Figure 6:**
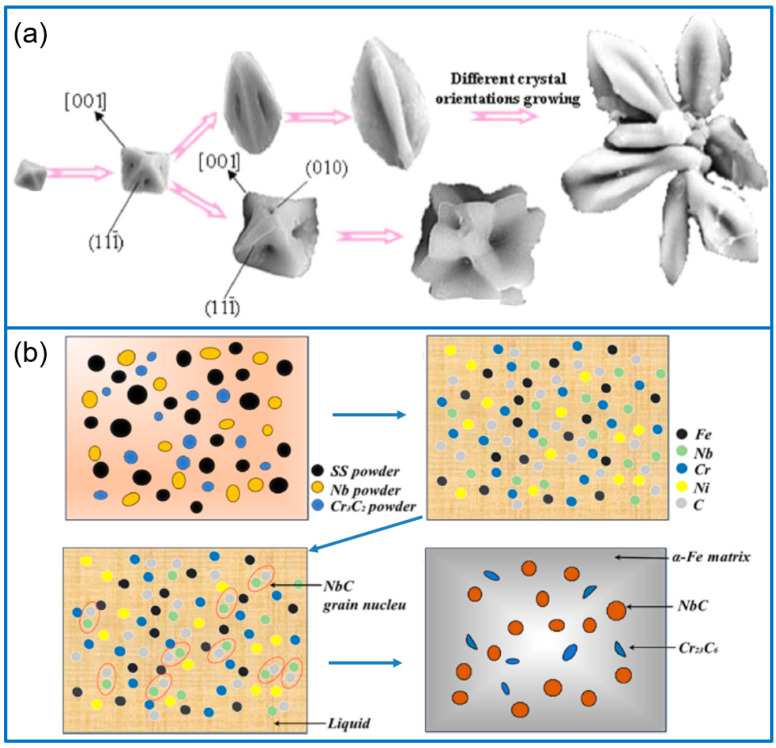
Formation schemes of two typical NbC petals (**a**) [65]; Copyright (2016) Elsevier. Illustration for laser cladding process of I-NbC composite coating (**b**) [67]. Copyright (2024) Elsevier.

Various NbC/Fe composite coatings have been fabricated mostly by in situ synthesis methods using different reactants. For example, by adding the mixed powders of C, Nb, and Cr_3_C_2_, different morphologies of NbC particles were in situ-generated for enhancing the wear resistance of Fe-based coating [64]. By adjusting the ratio of raw materials, the products of Cr_23_C_6_ and Cr_0.19_Fe_0.7_Ni_0.11_ compounds were formed at grain boundaries. Through comparison, cross-shaped NbC-reinforced coating exhibited a better wear resistance than that of rectangular NbC. In addition, Cao et al. [65] also prepared in situ NbC-modified Fe-based coating. In their work, various NbC phases were generated, including particles, dendrites, polyhedrons, networks, and petals (Figure 6a). The result demonstrated that the morphology of NbC had no great effect on the coating microhardness, while an increasing NbC fraction would enhance the coating microhardness. Chen et al. [66] fabricated in situ NbC-reinforced Fe-based coating by using Nb and B4C powders and disclosed the in situ synthesis mechanism by combining experiment and simulation. The coating composition was composed of [Fe-Cr] solid solution and Fe_2_B, NbC, and B_4_C-reinforced phases. During the cladding process, NbC particles (1.03 μm) were in situ-generated at the grain boundary and played a fine-grained strengthening effect. Compared with Fe-based coating, the reinforced coating showed a higher hardness (4.16 times) and lower volume loss (reduced 5 times). Among the literature about NbC-reinforced Fe-based coatings, several works discuss the influence of the addition method on coating performance. To solve this problem, Zhang et al. [67] fabricated NbC/Fe composite coatings on SS304 substrate by direct addition and in situ synthesis method, and the sample was denoted as A-NbC and I-NbC, respectively. Different from A-NbC, the I-NbC coating achieved a fine dispersion of the reinforcement phase, as shown in Figure 6b. The microhardness of I-NbC and A-NbC coating was 534.9 ± 4 HV and 524.6 ± 12 HV. However, the I-NbC coating had a minimum wear rate and friction coefficient, and the enhanced wear resistance was ascribed to the generation of passivation film by introducing Cr and Nb atoms.

### 4.3. NbC/HEAs Composite Coatings

CrMnFeCoNi high-entropy alloys (HEAs) with a face-centered cubic (FCC) feature have received widespread attention, due to their good fracture toughness and plasticity. To further enhance the mechanical properties of CrMnFeCoNi HEAs, Sun et al. [68] fabricated ceramic particles enhanced CrMnFeCoNi coating by using the powders of B4C, TiC, or NbC. During the laser cladding process, TiC and NbC were stably dispersed in the CrMnFeCoNi matrix, while the metal matrix reacted with B_4_C to produce M_2_B and M_23_C_6_ phases. The result confirmed that the coating containing NbC nanoparticles exhibited a better wear resistance than that of the coating reinforced by TiC, due to the mechanism of dislocation reinforcement and dispersion reinforcement. In this respect, Wu et al. [69] prepared NbC-reinforced FeNiCoCr HEAs coatings on the 316 SS, and discussed the influence of NbC content on the corrosive wear performance and corrosion resistance. Through comparison, the coating of FeNiCoCr + 10 wt% NbC possessed a superior corrosion resistance, and the corrosive wear rate was as low as 1.89 × 10^−5^ mm^3^/Nm. The coating microhardness was 440.8 HV_0.2_, which was greatly enhanced than that with no NbC.

To sum up, NbC-reinforced composite coatings are highlighted in this section. Among the existing metal matrices, NbC-reinforced Ni-based, Fe-based, and HEAs coatings have been widely reported in recent years. Regarding the preparation method, NbC-based coatings are usually fabricated by in situ synthesis method, and the raw materials usually include Nb, C, Nb, Cr_3_C_2_, B_4_C, or other carbides. Due to the high melting point and high hardness of NbC, the addition of NbC in the composite coating would significantly enhance the hardness, wear resistance, and corrosion resistance. To date, the number of documents about NbC-reinforced laser cladding coatings is much less than that of WC- and TiC-based composite coating.

## 5. Ti_n+1_AlC_n_ (MAX Phase) Reinforced Composite Coatings

MAX phase (M_n+1_AX_n_, *n* = 1–3) exhibits a typical nano-laminated structure [70], in which, X is a C or N element, A is a metal element of III–IV_A_, M is a Ti element. Nano-layered MAX phases integrate the typical characteristics of ceramic and metal, and present a superior wear resistance, corrosion resistance, good thermal conductivity, and oxidation resistance. When X is a C element, Ti_2_AlC or Ti_3_AlC_2_ belonged to bimetallic carbides. The nano-layered structure of MAX enables them an excellent self-lubricating property. Thus, serving as reinforced phases, Ti_2_AlC and Ti_3_AlC_2_ have been used for preparing laser cladding coatings to improve corrosion resistance and wear resistance. Among the existing reports, various MAX-reinforced metal coatings including Fe-based, Ni-based, Co-based, and Ti-based composite coatings have been developed. Based on the feeding method, direct addition and in situ synthesis of MAX phases are classified.

### 5.1. Direct Addition of MAX Phases

**Figure 7:**
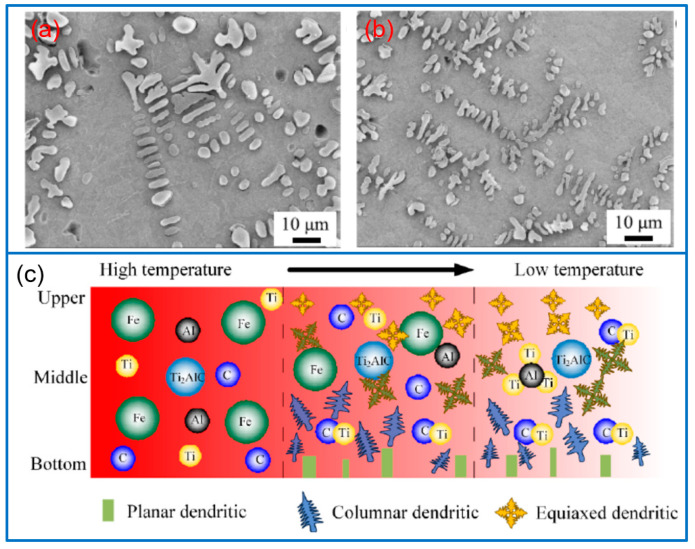
Morphologies of the sample of CoNi-50Ti_2_AlC (**a**) and CoNI-50Ti_3_AlC_2_ (**b**) [74]; Copyright (2024) Elsevier. The reaction process of Ti_2_AlC in the melting pool (**c**) [75]. Copyright (2024) Elsevier.

Adopting Ti_3_AlC_2_ or Ti_2_AlC as a reinforced phase, Rui et al. [71] prepared three kinds of Ni60-xTi_3_AlC_2_ (x = 4%, 8%, and 12%) composite coatings on S355 steel and disclosed the influence of Ti_3_AlC_2_ content on corrosion electrochemical performance of the coating. The composition of the coating was TiC, Al_4_C_3_, Ti_3_AlC_2_, Ti_x_Al_y_, and Al_3_Ti phases. Through comparison, the Ni60-12%Ti_3_AlC_2_ coating showed the optimal salt spray corrosion resistance, due to the generation of TiO_2_ and α-Al_2_O_3_ oxide film. Zhang et al. [72] investigated the influence of different Ti_2_AlC fractions on the structure and performance of Ni60/WC/xTi_2_AlC composite coatings. The coating composition was TiC, Al_2_O_3_, WC, and W_2_C phases, which exhibited irregular bulk and dendritic structures. The increased Ti_2_AlC fraction would refine the grain size and enhance the hardness. The coating containing 15%Ti_2_AlC presented the smallest friction coefficient and wear rate. Moreover, the addition of Ti_2_AlC also improved the tribocorrosion property and abrasive wear performance. In addition, Ti_3_AlC_2_ enhanced Co-based coatings were cladded on the surface of H13 steel [73], and the effect of Ti_3_AlC_2_ fraction on the structure and friction-wear property was investigated. The result implied that the Co-5%Ti_3_AlC_2_ coating possessed the highest hardness and the lowest wear rate. The superior wear resistance was attributed to the enhanced oxidation resistance derived from Al-generated Al_2_O_3_ film, and the self-lubrication feature of Ti_3_AlC_2_. By using the raw materials of Ti_3_AlC_2_, Ti_2_AlC, Co, and Ni powders, Lu et al. [74] fabricated CoNi-50Ti_3_AlC_2_ and CoNi-50Ti_2_AlC coatings on the surface of Ti6Al4V alloy to improve the tribological property. Different compositions were formed in the two coatings by using different MAX materials, as shown in Figure 7a,b. The TiAl_3_ and Ti_8_C_5_ phases were generated in the coating with Ti_2_AlC. While TiAl_3_, Ti_8_C_5_, and TiC phases were generated from Ti_3_AlC_2_. Compared with the decomposition of Ti_8_C_5_ in CoNi-50Ti_2_AlC coating, Ti_8_C_5_ phases remained in CoNi-50Ti_3_AlC_2_ coating, further enhancing the wear resistance.

In addition to the direct addition of Ti_2_AlC powder, Zhou et al. [76] prepared core-shell structured Ti_2_AlC@Ni powder for fabricating Ni-based cermet coating by high-speed laser cladding method. During the preparation process, the Ti_2_AlC phase decomposed into Al_2_O_3_, and TiC phases dispersed in the coating. Benefiting from the grain refinement, dispersion strengthening, and solid solution strengthening, the coating hardness was 2.8 times, and the wear resistance was 8.2 times (5 N load) that of the substrate. In addition, Jing et al. [75] also adopted Ni-coated Ti_2_AlC powders (Ti_2_AlC-Ni) for fabricating Fe-Ti_2_AlC-Ni coatings on the substrate of Cr_12_MoV. When the fraction of Ti_2_AlC was between 5 and 10 wt%, the coatings had no cracks, with a good surface. The reaction process was provided in Figure 7c, and the coating composition was verified as Ti_3_Al, TiC hard phases, and Ti_2_AlC self-lubrication phase. The in situ formation of hard phases enhanced the coating hardness. Through comparison, the Fe-based coating with 10 wt% Ti_2_AlC exhibited the lowest wear rate and frictional coefficient.

Different from the direct addition of Ti_2_AlC and metal powders, Hua et al. [77] fabricated TC4/Ti_2_AlC/B composite coating on Ti6Al4V (TC4) alloy by adding B powder. Moreover, the ultrasonic impact treatment (UIT) was conducted to further enhance the coating performance. The composition of the coating was verified as TiC, TiB, Ti3Al, Ti_2_AlC phase, Fe-Ti-V, and α-Ti solid solutions, and the composition had no change with different B contents and UIT. The addition of ceramics phases greatly enhanced the microhardness of the coating. The optimized coating with 6 wt% B/Ti_2_AlC/TC4 and UIT exhibited the lowest friction coefficient and the best wear resistance, due to the synergistic effect of TiO_2_, Al_2_O_3_ oxide films, and Ti_2_AlC lubricant, as well as the enhanced compressive stress by UIT.

### 5.2. In Situ Synthesis of MAX Phases

The in situ synthesis of Ti_2_AlC (MAX) phases is different from the direct addition method, and the elements of Ti, Al, and C must be added to generate MAX phases, and the ratio of different components should be considered. For example, Richardson et al. [78] synthesized Ti-Al-C MAX phase coatings on Ti substrate by adding the Ti/Al/C powder with a stoichiometric ratio of 2/1/1. The surface composition of the coating was TiC_0.64_, Ti_2_AlC, Ti_3_Al, and TiAl phases. The maximum hardness of the sub-surface region reached 811 ± 11 HV. In addition, Richardson et al. [79] also revealed the effect of thermal annealing on the Ti-Al-C system in laser-clad coatings. In their work, a furnace annealing at 1350 °C was conducted to induce the formation of large-scale MAX phase coatings. The result indicated that the reaction between α-Ti supersaturated with 33 at% C, Ti_y_Al_z_, and TiC_x_ generated dendritic Ti_2_AlC phase grains.

By adding TiC, TiN, and TiAl mixed powders, Sun et al. [80] fabricated Ti-Al-C-N protective coatings on the surface of the Ti6Al4V alloy, and confirmed the in situ generation of the MAX phase in the coating. By adjusting the TiC/TiAl/TiN ratio, the coating with the TiC/TiAl/TiN ratio of 1/1/1 presented the best combination performance. The maximum hardness of the coating was 712–950 HV, about twice the substrate. Furthermore, the coating also exhibited the best fretting wear resistance. The enhanced wear resistance was ascribed to the strengthening role of twin Ti_2_AlC/Ti_2_AlN phases, with no serious stress concentration. Based on the raw materials of TiC powder, Al powder, and TiAl alloy powder, Tian et al. [81] synthesized Ti-Al-C MAX phase reinforced coatings by laser cladding and post-laser treatment. During the laser cladding process, a core-shell structure consisting of the Ti_2_AlC shell and TiC core was generated. The late treatment promoted the diffusion of Al and C atoms, further inducing the nucleation-growth of the Ti_2_AlC phase to form MAX phase structures. The post-treatment greatly enhanced the content of the MAX phase in the coating, about 9.03 times higher than that in the cladded coating. The post-laser treatment led to the uniform distribution of microhardness in the coating, and the average microhardness reached 467.5 HV. In another work, the TiC/TiAl mixed powders were used to prepare MAX phase coatings on the substrate of Ti6Al4V alloys [82]. The composition of the coating was Ti_2_AlC, TiC, Ti-Al compounds, and a few Ti_3_AlC phases. An increased TiC fraction would increase the content of the resulting Ti_2_AlC phase, while Al powders improved the quality of the coating. Compared with the Ti alloy substrate, the hardness and wear resistance of the Ti-Al-C composite coating were significantly improved.

In addition to the formation of Ti-Al-C, other metal-reinforced phases can be introduced. For example, by using the mixed powders of Ti4822, Nb, and TiC, Wu et al. [83] fabricated Ti-Al-C-Nb_x_ composite coatings on TC4 alloy substrate, and investigated the influence of Nb fraction on the structure and tribological performance of the composite coating. Nb fraction greatly affected the microstructure of the coating, further affecting the coating performance. A high Nb fraction would induce the decomposition of Ti_3_AlC_2_ to produce TiC and Ti_2_AlC, due to the introduction of Nb atoms into the Ti_3_AlC_2_ phase. Through comparison, the coating with 2 at% Nb presented the smallest wear rate and friction coefficient.

Serving as reinforced phases, bimetallic carbides Ti_2_AlC and Ti_3_AlC_2_ belong to the typical MAX phases, which combine the merits of ceramic phases and metal, as well as the typical 2D nano-layered structure. The purpose of adding MAX phases is to improve the hardness and wear resistance of the coating. In this section, the research progress of various MAX-reinforced metal coatings has been introduced. Among the literature about the direct addition of MAX, the researchers majorly focus on the influence of MAX content, the difference between Ti_3_AlC_2_ and Ti_2_AlC, the surface Ni wrapping of Ti_2_AlC, or introduction of the B element. While for the works about in situ generation of MAX, the research hotspots mostly focus on the selection of raw materials and their ratio, such as Ti/Al/C mixed powders, TiC/Al/TiAl alloy powder, and TiC/TiN/TiAl powder. The resulting MAX products are different from the direct addition of MAX. In addition to the MAX phases, other products are introduced in the coating, which is determined by the raw materials. In order to obtain MAX phase product in the composite coatings, some researchers have adopted thermal annealing treatment to induce the generation of MAX phases. Compared with WC, TiC, and NbC, the Ti_2_AlC- and Ti_3_AlC_2_-reinforced coatings are more complicated, and the problems involve the universality of raw materials, the uncertainty of MAX substance, and the formation of multi-component products.

## 6. Cr_3_C_2_, TaC-Reinforced Composite Coatings

### 6.1. Cr_3_C_2_-Reinforced Coatings

**Figure 8:**
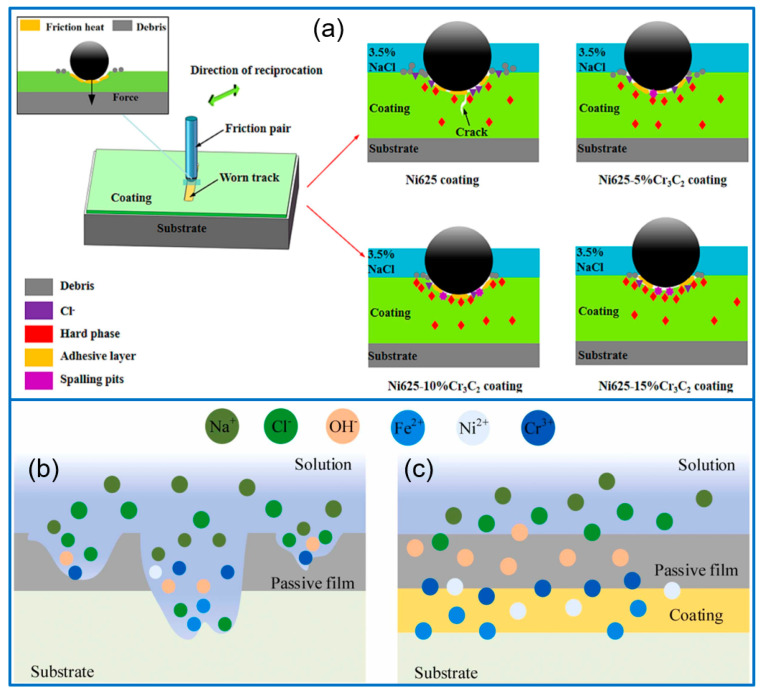
Wear models on Ni625−xCr_3_C_2_ coatings in 3.5% NaCl solution (**a**) [86]; Copyright (2024) Elsevier. The schematic diagram of the corrosion mechanism of pure alloy (**b**) and composite coating (**c**) [87]. Copyright (2024) Elsevier.

Cr_3_C_2_ ceramic powders exhibit some advantages of superior oxidation resistance, high hardness, and corrosion resistance, which have served as strengthening phases for enhancing the mechanical performance and wear resistance of different metal alloys. Cr_3_C_2_/Ni composite coatings have been widely developed. In this respect, Su et al. [84] deposited Ni60A/Cr_3_C_2_ composite coating on the substrate of 60Si_2_Mn steel and discussed the influence of process parameters. The priority order of coating quality was scanning speed, feeding rate, and laser power. The coating composition was NiO, γ-Ni, M_23_C_6_ and M_7_C_3_. The optimized T6 coating was prepared with 5 mm/s scanning speed, 1800 W laser power, and 8 g/min feeding rate, which presented a high hardness of 902 HV_0.1_, with enhanced wear resistance. In addition, the Cr_3_C_2_-reinforced Ni60A-Ag self-lubricating coating was cladded on a Cu alloy substrate [85]. Ag particles and Cr_7_C_3_ dendrites were dispersed in the γ-Ni matrix uniformly, further improving the hardness and plastic deformation resistance. Moreover, the friction coefficient of the coating was less than 0.25, much lower than that of Cu alloy. Similar to Ni60A/Cr_3_C_2_ composite coating, Ni625-xCr_3_C_2_ coatings were also cladded on the substrate of C45E4 steel [86]. The hardness of composite coating increased with an increasing Cr_3_C_2_ content. Moreover, the wear rate and friction coefficient were greatly reduced compared with pure Ni625 coating. The Ni625-10%Cr_3_C_2_ coating exhibited optimal corrosion resistance, and the corresponding mechanism was presented in Figure 8a.

In addition to the Ni-based composite coating, Cr_3_C_2_-reinforced Fe-based and other alloys have been reported. For example, Mao et al. [87] developed Cr_3_C_2_/15-5PH stainless steel composite coatings by laser cladding. The introduction of Cr_3_C_2_ induced the phase transition from α-Fe to γ-Fe and produced new phases of M_7_C_3_ and Cr_23_C_6_. The composite coating containing 15 wt% Cr_3_C_2_ presented a superior corrosion resistance in 3.5 wt% NaCl solution, as given in Figure 8b,c. In addition, the Cr_3_C_2_/Fe_3_Al composite coating was cladded on carbon structural steel to improve the hardness and wear resistance [88]. Fan et al. [89] prepared Cr_3_C_2_-reinforced Ni_3_Al coatings and discussed the effect of Cr_3_C_2_ content on the structure, mechanical properties, and friction behaviors of the coating. The introduced Cr_3_C_2_ enhanced the coating toughness and hardness, as well as the wear resistance. Through comparison, the coating with 15 wt% Cr_3_C_2_ presented the minimum friction coefficients and wear rate. Aghili et al. [90] fabricated NiCr-Cr_3_C_2_ coating on the TiAl substrate by cladding the powder mixture and investigated the oxidation behavior of coating at 900 °C. The XRD result confirmed that the coating consisted of γ solid solution containing Cr_7_C_3_, Cr_3_C_2_, and Cr_23_C_6_ phases. Compared with the TiAl substrate, the NiCr-Cr_3_C_2_ coating presented a better oxidation resistance, due to the formation of an oxide surface layer. In addition, the authors also discussed the influence of processing parameters on coating properties and microhardness [91]. The microhardness of optimized NiCr-Cr_3_C_2_ coating reached 820 HV, much higher than that of pure TiAl substrate (320 HV). Venkatesh et al. [92] prepared Cr_x_C_y_-NiCrMoNb composite coatings on the surface of SA516 steel. In their experiment, Cr_7_C_3_ and Cr_3_C_2_ acted as starting materials, and only Cr_7_C_3_ dendrites were found in the cladding coating, indicating that Cr_7_C_3_ was in a stable phase during the process. The increasing scanning speed and laser power greatly decreased the fraction of carbides in the coating. The lower carbide content would induce a decrease in coating hardness, while the fine hard phase spacing and solid solution strengthening would cause an increase in hardness.

### 6.2. TaC-Reinforced Coatings

TaC has a high hardness of 1550 HV, a high melting point of 3980 °C, and low resistance, which can be used for fabricating composite coatings with a good wear resistance and corrosion resistance. Unlike those traditional metal carbides reported above, there are few reports about TaC-reinforced metal coatings. Herein, TaC-reinforced metal coatings are introduced, including the direct addition of TaC, and in situ-generated TaC.

Li et al. [93] prepared TaC/Stellite X-40 Co-based composite coatings on the nickel-aluminum bronze (NAB) substrate. The resulting coating contained Cr_3_C_2_, TaC, and Co_3_Ta phases in the γ-Co matrix, further improving the electrochemical corrosion and wear resistance. The optimized coating containing 20 wt% TaC showed the highest hardness of 771.7 HV_0.2_, the minimum friction coefficient and wear rate, as well as a high self-corrosion potential. Except for adding TaC powders, TaC can be in situ synthesized in different metal matrices. For example, Yu et al. [94] prepared TaC/Ni-Al-Cr composite coatings by using Ta, C, and Ni-Al-Cr powders, which, in situ-generated TaC and enhanced the bonding strength between the matrix and TaC. The composite coating had a better wear resistance than that of Ni-Al-Cr coating. By using the raw materials of Ni, Ta powder, TiC, and Ni-coated graphite, Hu et al. [95] developed Ni_3_Ta-TaC-reinforced Ni-based coatings on 5Cr_5_MoSiV steel. The addition of TiC powder accelerated the in situ formation of TaC and Ni_3_Ta, and TaC particles were generated around TiC, which avoided the formation of cracks and Ni_3_Ta intermetallic compounds. The wear resistance of the composite coating was 4 times higher than the substrate. The authors also discussed the difference between Ni-Ta coating and Ni-Ta-C coating [96]. The composition of Ni-Ta-C coating was Ni_3_Ta, γ-Ni, and TaC phases. Due to the generation of TaC and Ni_3_Ta reinforced phases, the hardness and wear resistance of Ni-Ta-C coating were both better than that of Ni-Ta coating. Liu et al. [97] prepared Ta-reinforced cobalt-based coatings by using Ta powder and Stellite F cobalt-based alloy powder. The in situ-generated TaC phases reduced the impact of particles in the erosion process, further enhancing the erosion resistance. The coating containing 30 wt% Ta presented the best comprehensive performance.

To sum up, Cr_3_C_2_, TaC-reinforced composite coatings have been introduced in this part. Various Cr_3_C_2_-reinforced composite coatings have been investigated, and the topics involve the effect of process parameters and Cr_3_C_2_ content, the synergistic effect with Ag particles, and the selection of metal matrices, such as Ni, Fe, and different alloys. Among the existing literature, most of the Cr_3_C_2_-based coatings are fabricated by direct addition method, instead of in situ synthesis. However, TaC is usually in situ synthesized in different metal matrices by using the raw materials of Ta, C, and metal powders. The in situ-generated TaC phases greatly improve the coating hardness, wear resistance, and erosion resistance.

## 7. Conclusions and Prospects

### 7.1. Conclusions

The use of laser cladding technique to prepare metal carbide-reinforced coatings overcomes the difficult processing problem of ceramic materials, which can obtain composite coatings with an excellent performance in working conditions. Especially after adding different carbide-reinforcing agents, the hardness, wear resistance, corrosion resistance, and high-temperature oxidation resistance of the substrate can be significantly improved. Therefore, metal carbide-reinforced laser cladding coatings have important research and practical application value. According to the type of metal carbides, five sections are classified in this review, including WC-reinforced coatings, TiC-reinforced coatings, NbC-reinforced coatings, Ti_n+1_AlC_n_ (MAX) reinforced coatings, and Cr_3_C_2_, TaC-reinforced coatings. The selection of raw materials, fabrication method, microstructure/composition, and application performance of various carbide-reinforced composite coatings are highlighted in this review. The application of various metal carbides and performance enhancement of carbide-reinforced coatings are summarized. From Table 1, the application range, performance advantages and caution of each metal carbide can be easily obtained, which will help researchers to choose a suitable reinforcing phase for fabricating high-performance laser cladding coatings. In addition to the summary in Tables, some important conclusions are also drawn and listed as follows:

(1) As a typical metal carbide reinforcing agent, WC-reinforced coatings have been extensively reported, including WC/Ni, WC/Co, WC/Fe, and WC/other metal composite coatings. The research topics mostly focus on the effect of WC content and particle size, the optimization of process parameters, coating structure design, a combination of WC and other ceramic phases or oxides, the in situ synthesis of WC, the selection of metal matrix, and so on. The main function of WC in composite coatings is to enhance the hardness, mechanical properties, wear resistance, and corrosion resistance.
(2) Various TiC-reinforced composite coatings are introduced, including TiC/Ni, TiC/Fe, TiC/HEAs, and TiC/Ti alloy composite coatings. The research topics mostly involve the in situ synthesis of TiC by selecting different raw materials, such as Ti powers, nickel-coated graphite, B_4_C, Cr_3_C_2_, or WC. Different from the direct addition of TiC particles in a metal matrix, in situ-generated TiC would refine the grain size by adjusting the nucleation rate. Furthermore, the function of solid solution strengthening and second phase strengthening further enhances the coating hardness and wear resistance.
(3) NbC-reinforced Ni-based, Fe-based, and HEAs coatings have been developed in recent years. Compared with WC- and TiC-based composite coating, the amount of literature about NbC-reinforced composite coatings is much less. In terms of processing method, NbC-reinforced composite coatings are usually prepared by in situ synthesis method, and the powders of Nb, C, Nb, Cr_3_C_2_, B_4_C, or other carbides are adopted as raw materials. The high melting point and high hardness of NbC enable the NbC-reinforced composite coatings with a high hardness, wear resistance, and corrosion resistance.
(4) Bimetallic carbides Ti_2_AlC and Ti_3_AlC_2_ belong to MAX phases with a typical 2D nano-layered structure. By using MAX as fillers, the researchers have discussed the effect of MAX dosage, the difference between Ti_3_AlC_2_ and Ti_2_AlC, the surface wrapping, and the introduction of other nonmetal elements. While for the works about in situ generation of MAX, the researchers pay more attention to the selection of raw materials and the optimization of component ratio. The in situ-generated MAX products are different from the direct addition of MAX, and thermal annealing treatment is required to induce the formation of MAX phases. The issues about MAX reinforced coatings are related to the universality of raw materials, the uncertainty of MAX substance, and the formation of multi-component products.
(5) In respect to Cr_3_C_2_-reinforced composite coatings, researchers have discussed the effect of process parameters and Cr_3_C_2_ content, the synergistic effect with Ag particles, and the selection of metal matrices, such as Ni, Fe, and different alloys. Most Cr_3_C_2_-based coatings are prepared by direct addition of Cr_3_C_2_. TaC-reinforced coatings are mostly prepared by in situ synthesis by using the mixed powders of Ta, C, and metal powders or other metal carbides. The introduction of TaC phases significantly improves the hardness, wear resistance, and erosion resistance of coatings.

### 7.2. Prospects

Although significant research achievements and progress have been achieved in the field of laser cladding coatings in recent years, there are still many issues that require further in-depth study and improvement.

(1) The selection of metal carbides in practical application and optimization design. Metal carbides have diversity, and the suitable selection of one or several carbides is really important. Moreover, the molding method including direct addition or in situ synthesis determines the industrialization process of composite coatings. Therefore, researchers must be familiar with the advantages/disadvantages and functionality of each metal carbide, and choose the appropriate metal carbide for the practical requirement.
(2) Defects such as pores and cracks may occur during the actual processing of laser cladding carbide reinforced coatings. The defects generated in composite coatings are generally related to the type, dosage of the filler, and processing parameters. Therefore, the researchers should select appropriate addition amounts and processing parameters, based on the physical feature, and particle size of various carbide fillers.
(3) The design and preparation of multi-functional ceramic coating materials, that is, how to add one or several metal carbides to prepare coatings with multiple excellent properties. This requires researchers to be familiar with the compatibility between metal substrates and carbides, and to select appropriate reinforcing agents based on the type of substrate. For example, a strong tough coating can be obtained by combining MAX-phase ceramics with metals. If the conditions permit, theory calculation results are required to guide the selection of metal carbides.

## Figures and Tables

**Table 1:** The application of various metal carbides in laser cladding coatings.

Carbides	Metal Matrix	Performance Enhancement	Caution
WC	Reinforced Ni, Co, Fe, various alloys	Enhancing the hardness, mechanical performance, wear/corrosion resistance	WC content is <20 wt%
TiC	Reinforced Ni, Fe, Ti, HEAs	Enhancing the hardness, wear/corrosion resistance; in situ-generated TiC is better	When TiC content is >15%, pores and microcracks
NbC	Reinforced Ni, Fe, HEAs	Enhancing the hardness, wear/corrosion resistance	Compared with direct addition, in situ NbC is better
Ti_n+1_AlC_n_	Reinforced Ni, Co, Fe, Ti	2D nano-layered structure, enhancing the hardness, wear/corrosion resistance	Require thermal annealing to produce MAX phases
Cr_3_C_2_	Reinforced Ni, Fe, TiAl	Enhancing the hardness, wear/corrosion/oxidation resistance	Cr_7_C_3_ was stable phase, much stabler than Cr_3_C_2_
TaC	Reinforced Ni, Co	Enhancing the hardness, wear/corrosion resistance	In situ formation of TaC avoids the cracks

## Data Availability

Not applicable.

## References

[1] Wang K., Zhang Z., Xiang D., Ju J. (2022). Research and Progress of Laser Cladding: Process, Materials and Applications. Coatings.

[2] Liu Y., Ding Y., Yang L., Sun R., Zhang T., Yang X. (2021). Research and progress of laser cladding on engineering alloys: A review. J. Manuf. Process..

[3] Wu Q., Long W., Zhang L., Zhao H. (2024). A review on ceramic coatings prepared by laser cladding technology. Opt. Laser Technol..

[4] Das A.K. (2022). Effect of rare earth oxide additive in coating deposited by laser cladding: A review. Mater. Today Proc..

[5] Zhu L., Xue P., Lan Q., Meng G., Ren Y., Yang Z., Xu P., Liu Z. (2021). Recent research and development status of laser cladding: A review. Opt. Laser Technol..

[6] Sun S., Wang J., Xu J., Cheng X., Jing C., Chen Z., Ru H., Liu Y., Jiao J. (2023). Preparing WC-Ni coatings with laser cladding technology: A review. Mater. Today Commun..

[7] Liu Y., Wang K., Fu H. (2023). Improvement of the High Temperature Wear Resistance of Laser Cladding Nickel-Based Coating: A Review. Metals.

[8] Liu D., Yang X., Zhao A., Cheng X., Zhang Q. (2024). Preparation of nickel-based composite coatings by laser cladding technology: A review. Int. J. Adv. Manuf. Technol..

[9] Wang H., Liu K., Li J., Geng S., Jing L., Skuratov V. (2024). Reinforcements/matrix micro-interface evolution and properties of in-situ Ni60A/WC coatings prepared by laser cladding. Surf. Coat. Technol..

[10] Hu Z., Li Y., Lu B., Tan N., Cai L., Yong Q. (2022). Effect of WC content on microstructure and properties of high-speed laser cladding Ni-based coating. Opt. Laser Technol..

[11] Liu Y., Gu X., Lou C., Kang L., Hou Q., Ma C. (2023). Influence of WC ceramic particles on structures and properties of laser cladding Ni50-WC coatings. J. Mater. Res. Technol..

[12] Li Y., Shi Y., Jiang G., Tang S., Wu J., Wang J. (2024). Effect of WC on microstructure, molten pool and properties of Ni based coatings by multi-layer laser cladding. Opt. Laser Technol..

[13] Shen X., He X., Gao L., Su G., Xu C., Xu N. (2022). Study on crack behavior of laser cladding ceramic-metal composite coating with high content of WC. Ceram. Int..

[14] Tan N., Hu Z., Zhou Y., Li Y., Lu B., Hu D., Liu Y., Li Q. (2024). Effect of WC particle size on the microstructure and tribological properties of high-speed laser cladding Ni/WC composite coatings. Mater. Today Commun..

[15] Wang Q., Li Q., Zhang L., Chen D.X., Jin H., Li J.D., Zhang J.W., Ban C.Y. (2022). Microstructure and properties of Ni-WC gradient composite coating prepared by laser cladding. Ceram. Int..

[16] Bian Y., Li S., Tian C., Chen B., Dong B., Shu Z., Guo J., He X., Yu G. (2025). Effect of powder feeding rate on mass fraction of WC particles, microstructure evolution and micro-hardness of WC-Ni composite coating by laser cladding. J. Alloys Compd..

[17] Li S., Huang K., Zhang Z., Zheng C., Li M., Wang L., Wu K., Tan H., Yi X. (2023). Wear mechanisms and micro-evaluation of WC + TiC particle-reinforced Ni-based composite coatings fabricated by laser cladding. Mater. Charact..

[18] Li M., Yang J., Han B., Song L., Li P., Dong W., Xue X. (2023). Comparative investigation on microstructures and properties of WC/Cr_3_C_2_ reinforced laser cladding Ni-based composite coatings subjected to ultrasonic impact treatment. Mater. Today Commun..

[19] Chen J., Lian G., Lin T., Lu H., Wang Y. (2024). Effects of the proportions of carbon on the microstructure and properties of NbC-reinforced Ni-WC composite coatings by laser cladding in-situ synthesis. Mater. Today Commun..

[20] Chen J., Lian G., Feng M., Zhang W., Chen R. (2025). Microstructure evolution and properties of (Nb,M)C (M=Ti,V and Zr) reinforced Ni-WC coatings by laser cladding. J. Alloys Compd..

[21] Zhao J., Li R., Feng A., Feng H. (2024). Effect of rare earth La_2_O_3_ particles on structure and properties of laser cladding WC-Ni60 composite coatings. Surf. Coat. Technol..

[22] Jiang C., Zhang J., Chen Y., Hou Z., Zhao Q., Li Y., Zhu L., Zhang F., Zhao Y. (2022). On enhancing wear resistance of titanium alloys by laser cladded WC-Co composite coatings. Int. J. Refract. Met. Hard Mater..

[23] Lv N., Yue H., Guo C., Dai W., Zhang J., Li Q., Zhao G., Hao G. (2024). A comparative investigation on the effects of reinforcement phase addition methods on laser melting deposited WC/Co coatings. J. Manuf. Process..

[24] Xu P., Zhu L., Xue P., Yang Z., Wang S., Ning J., Meng G., Lan Q., Qin S. (2022). Microstructure and properties of IN718/WC-12Co composite coating by laser cladding. Ceram. Int..

[25] Ding H., Cao Y., Hua K., Tong Y., Li N., Sun L., Li X., Wu H., Wang H. (2023). Fretting wear resistance at ambient and elevated temperatures of 316 stainless steel improved by laser cladding with Co-based alloy/WC/CaF_2_ composite coating. Opt. Laser Technol..

[26] Xiao Q., Sun W.L., Yang K.X., Xing X.F., Chen Z.H., Zhou H.N., Lu J. (2021). Wear mechanisms and micro-evaluation on WC particles investigation of WC-Fe composite coatings fabricated by laser cladding. Surf. Coat. Technol..

[27] Li J., Zhu Z., Peng Y., Shen G. (2021). Phase evolution and wear resistance of in-situ synthesized (Cr, W)_23_C_6_-WC composite ceramics reinforced Fe-based composite coatings produced by laser cladding. Vacuum.

[28] Zeng X., Wang Q., Chen C., Lian G., Huang X. (2021). Effects of WC addition on the morphology, microstructure and mechanical properties of Fe50/TiC/WC laser claddings on AISI 1045 steel. Surf. Coat. Technol..

[29] Yang Z., Hao H., Gao Q., Cao Y., Han R., Qi H. (2021). Strengthening mechanism and high-temperature properties of H13 + WC/Y_2_O_3_ laser-cladding coatings. Surf. Coat. Technol..

[30] Liu J., Li Z., Lin D., Tang Z., Song X., He P., Zhang S., Bian H., Fu W., Song Y. (2024). Eutectic high-entropy alloys and their applications in materials processing engineering: A review. J. Mater. Sci. Technol..

[31] Li Z., Xie D., Liu Y., Lv F., Zhou K., Jiao C., Gao X., Wang D., Liu Y., Zu H. (2024). Effect of WC on the microstructure and mechanical properties of laser-clad AlCoCrFeNi_2.1_ eutectic high-entropy alloy composite coatings. J. Alloys Compd..

[32] Du M., Wang L., Gao Z., Yang X., Liu T., Zhan X. (2022). Microstructure and element distribution characteristics of Y_2_O_3_ modulated WC reinforced coating on Invar alloys by laser cladding. Opt. Laser Technol..

[33] Cai Q., Li G., Wu B., Xu S., Wang L., Guo Y. (2024). Effect of TiC content on microstructure and properties of TiC/Ni60 coatings on Ti6Al4V alloy deposited by laser cladding. Opt. Laser Technol..

[34] Meng Q., Wang C., Liu T., Song Q., Xue B., Cui H. (2024). Microstructure and performance optimization of laser cladding nano-TiC modified nickel-based alloy coatings. Surf. Coat. Technol..

[35] Chen L., Yu T., Chen X., Zhao Y., Guan C. (2022). Process optimization, microstructure and microhardness of coaxial laser cladding TiC reinforced Ni-based composite coatings. Opt. Laser Technol..

[36] Chen L., Chen Y., Chen X., Yu T., Wang Z. (2022). Microstructure and properties of in situ TiC/Ni functionally gradient coatings by powder-fed laser cladding. Ceram. Int..

[37] Chen L., Guan C., Ma Z., Cui Z., Zhang Z., Yu T., Gu R. (2025). Modeling and simulation of grinding surface morphology for laser cladding in situ TiC reinforced Ni-based composite coatings. Surf. Coat. Technol..

[38] Wang H., Wu M., Miao X., Jin X., Cui C. (2024). TiC in-situ strengthening mechanism and crack initiation mechanism of SiC/TC4 composite coatings by laser cladding. Ceram. Int..

[39] Qi X., Li Y., Cui W., Du J., Zhao Y., Li F. (2025). Improving the wear and corrosion properties of laser cladded Ni-based composite coatings via regulating in-situ TiB_2_-TiC. Ceram. Int..

[40] Cui W., Li Y., Li F., Qi X., Sun X., Pan Z., Niu J. (2024). Wear and corrosion properties of in-situ TiC–TiB_2_ modified Ni-based composite coatings with different B/C ratios prepared by laser cladding. Ceram. Int..

[41] Zheng Y., Lian G., Lu H., Chen C., Huang X. (2024). Defects, organization, and properties of TiB_2_–TiC Bi-ceramic phase by laser cladding in situ synthesis. Ceram. Int..

[42] Saeedi R., Shoja Razavi R., Bakhshi S.R., Erfanmanesh M., Ahmadi Bani A. (2021). Optimization and characterization of laser cladding of NiCr and NiCr–TiC composite coatings on AISI 420 stainless steel. Ceram. Int..

[43] Ge T., Chen L., Gu P., Ren X., Chen X. (2022). Microstructure and corrosion resistance of TiC/Inconel 625 composite coatings by extreme high speed laser cladding. Opt. Laser Technol..

[44] Zhu H., Ouyang M., Hu J., Zhang J., Qiu C. (2021). Design and development of TiC-reinforced 410 martensitic stainless steel coatings fabricated by laser cladding. Ceram. Int..

[45] Hui X., Dejun K. (2024). Microstructure and tribological properties of laser cladded TiC reinforced WC-10Co4Cr coatings at 500 °C. Mater. Today Commun..

[46] Zhang H.F., Wang L., Zhang S., Wu C.L., Zhang C.H., Sun X.Y. (2023). Design, fabrication, microstructure and properties of in-situ synthesized TiC reinforced stainless steel matrix composite coating by laser cladding. Mater. Charact..

[47] Yang X., Chen Y., Zhang Z., Liu Y., Tao S., Li G., Chen H. (2023). Study on microstructure and properties of laser-clad Fe-based (Ti, V)C composite coatings. Surf. Coat. Technol..

[48] Jiang G.Y., Zhang J.W. (2024). The influence of laser power on the microstructure and friction performance of laser-prepared TiC-NbC composite coatings on stainless steel surfaces. Mater. Today Commun..

[49] Chen L., Yu T., Guan C., Zhao Y. (2022). Microstructure and properties of metal parts remanufactured by laser cladding TiC and TiB_2_ reinforced Fe-based coatings. Ceram. Int..

[50] Chen G.D., Liu X.B., Yang C.M., Zhang F.Z., Li X.G., Zheng J., Liu J. (2024). Strengthening mechanisms of laser cladding TiC/FeCoCrNiCu high-entropy composite coatings: Microstructure evolution and wear behaviors. Tribol. Int..

[51] Li Y., Wang K., Fu H., Guo X., Lin J. (2022). Microstructure and wear resistance of in-situ TiC reinforced AlCoCrFeNi-based coatings by laser cladding. Appl. Surf. Sci..

[52] Gao Z., Niu Z., Gao Z., Li J., Bai G., Ke L., Yu Y., Zhang C. (2024). Microstructure and wear behavior of in-situ synthesized TiC-reinforced CoCrFeNi high entropy alloy prepared by laser cladding. Appl. Surf. Sci..

[53] Li Y., Fu H., Ma T., Wang K., Yang X., Lin J. (2022). Microstructure and wear resistance of AlCoCrFeNi-WC/TiC composite coating by laser cladding. Mater. Charact..

[54] Li S., Niu W., Lei Y.W., Zheng Y. (2024). Microstructure and wear performance of ex/in-situ TiC reinforced CoCrFeNiW_0.4_Si_0.2_ high-entropy alloy coatings by laser cladding. J. Alloys Compd..

[55] Zhuang D.D., Tao W.W., Ni H.M., Wang A.Z., Du B., Zhang S.H., Lian X.L., Wang D., Feng Y.J. (2023). TiC distribution and properties of TiC-CrMnFeCoNi coating fabricated by laser cladding with ultrasound. Surf. Coat. Technol..

[56] Chen N., Xiao H., Ren L., Huang F., Chen Y., Cao S., Wu H., Zhu L. (2024). Microstructure and tribological properties of laser-cladded TiCx/TiAl composite coatings on TC4 alloy. Tribol. Int..

[57] Chen T., Li W., Liu D., Xiong Y., Zhu X. (2021). Effects of heat treatment on microstructure and mechanical properties of TiC/TiB composite bioinert ceramic coatings in-situ synthesized by laser cladding on Ti6Al4V. Ceram. Int..

[58] Zhang Z., Yang F., Zhang H., Zhang T., Wang H., Xu Y., Ma Q. (2021). Influence of CeO_2_ addition on forming quality and microstructure of TiCx-reinforced CrTi4-based laser cladding composite coating. Mater. Charact..

[59] Zhang Z., Yang F., Zhang H., Zhang T., Wang H. (2021). Microstructure and element distribution of laser cladding TiCx-reinforced CrTi4-based composite coating with CeO_2_/Ce_2_O_3_. Mater. Lett..

[60] Gaddam S., Nartu M.S.K.K.Y., Chesetti A., Mantri S.A., Mishra R.S., Dahotre N.B., Banerjee R. (2023). Hierarchical phase evolution during direct laser deposition of an in-situ Ni-NbC composite. Scripta Mater..

[61] Zhang Y., Yu T., Sun J., Sun Z., Wang Y. (2024). Effect of in-situ NbC content on the microstructure and mechanical properties of Ni625 composite coating by laser cladding. Ceram. Int..

[62] Lian G., Yue K., Chen C., Huang L., Zheng M. (2023). Influences of powder ratios on the mechanical properties of in-situ synthetic NbC coatings. Mater. Today Commun..

[63] Lian G., Chen J., Lu H., Chen C., Huang X. (2025). The microstructure and properties of reinforced Ni-20WC coatings by the laser-cladding in-situ synthesis of xNbC. Ceram. Int..

[64] Xi W., Song B., Sun Z., Yu T., Wang J., Sun Q. (2023). Effect of various morphology of in situ generated NbC particles on the wear resistance of Fe-based cladding. Ceram. Int..

[65] Cao Y., Zhi S., Gao Q., Tian X., Geng T., Guan X., Qin C. (2016). Formation behavior of in-situ NbC in Fe-based laser cladding coatings. Mater. Charact..

[66] Chen L., Yu T., Xu P., Zhang B. (2021). In-situ NbC reinforced Fe-based coating by laser cladding: Simulation and experiment. Surf. Coat. Technol..

[67] Zhang H.F., Zhang S., Wu H., Wang R., Zhang C.H., Wu C.L., Chen J., Chen H.T. (2024). Mechanical properties and corrosion resistance of laser cladding iron-based coatings with two types of NbC reinforcement. Surf. Coat. Technol..

[68] Sun D., Zhu L., Cai Y., Yan Y., Ge F., Shan M., Tian Y., Han J., Jiang Z. (2022). Tribology comparison of laser-cladded CrMnFeCoNi coatings reinforced by three types of ceramic (TiC/NbC/B_4_C). Surf. Coat. Technol..

[69] Wu H., Wang Z.Y., Wang M.S., Wang R., Zhang S., Zhang C.H., Wu C.L., Chen H.T., Chen J. (2025). Microstructure evolution, corrosion and corrosive wear properties of NbC-reinforced FeNiCoCr-based high entropy alloys coatings fabricated by laser cladding. Eng. Fail. Anal..

[70] Zhang Z., Duan X., Jia D., Zhou Y., van der Zwaag S. (2021). On the formation mechanisms and properties of MAX phases: A review. J. Eur. Ceram. Soc..

[71] Rui S., Chao Z., Dejun K. (2023). Salt spray corrosion behavior and electrochemical performance of laser cladded Ni60–Ti_3_AlC_2_ composite coatings in 3.5% NaCl solution. Mater. Today Commun..

[72] Zhang K., Kong D. (2025). Microstructure, tribocorrosion and electrochemical properties of Ti_2_AlC reinforced Ni60WC coatings by high–speed laser cladding. Mater. Charact..

[73] Zhou J., Kong D. (2021). Friction–wear performances and oxidation behaviors of Ti_3_AlC_2_ reinforced Co–based alloy coatings by laser cladding. Surf. Coat. Technol..

[74] Lu Y., Peng Y., Chang X., Kong D. (2024). Ti_2_AlC and Ti_3_AlC_2_ reinforced CoNi coatings by laser cladding: Nanostructures, tribological properties and density functional theory calculations. J. Manuf. Process..

[75] Jing S., Li Y., Cai Y., Tan N., Li Q., Zhang G., Li G. (2024). Investigation on microstructure and tribological properties of Ti_2_AlC-Ni reinforced Fe-based prepared by high-speed laser cladding. Surf. Coat. Technol..

[76] Zhou L., Ma G., Wang H., Wang W., Mou H., Zhu X., Zhao H., Li Y., Tan N. (2024). High-speed laser cladded Ni-based cermet coating with high ceramic phase content derived from core-shell structured powder. Surf. Coat. Technol..

[77] Hua S.W., Pang M., Ji F.Q., Chen J., Liu G. (2023). Microstructure and tribological properties of Ti_2_AlC-B particle-enhanced self-lubricating coatings on Ti6Al4V by ultrasonic impact treatment and laser cladding. Mater. Today Commun..

[78] Richardson P., Cuskelly D., Brandt M., Kisi E. (2020). Microstructural analysis of in-situ reacted Ti_2_AlC MAX phase composite coating by laser cladding. Surf. Coat. Technol..

[79] Richardson P., Cuskelly D., Brandt M., Kisi E. (2021). Effects of furnace annealing on in situ reacted Ti_2_AlC MAX phase composite coatings deposited by laser cladding. Surf. Coat. Technol..

[80] Sun L., Cao Y., Ding H., Wang Y., Ma Q., Hua K., Wang H. (2024). Fabrication of composite coatings containing in-situ reacted Ti_2_AlC/Ti_2_AlN MAX phases by laser cladding and investigation of fretting wear mechanism. Ceram. Int..

[81] Tian Y., Xiao H., Ren L., Feng J., Xiao Y., Chen N., Zhou X. (2022). A new strategy to fabricate Ti_2_AlC MAX coatings by the two-step laser method. Surf. Coat. Technol..

[82] Chu M., Xiao H., Ren L., Mo T., Lin B. (2024). Microstructure and properties of Ti–Al–C composite coatings prepared by laser cladding. Ceram. Int..

[83] Wu H., Xiao H., Chen N., Chu M., Lin B., Zhang Z., Fu G., Mo T. (2025). Effect of Nb content on the tribological properties of laser-cladded Ti-Al-C MAX phase composite coatings. Tribol. Int..

[84] Su Z., Li J., Shi Y., Ren S., Zhang Z., Wang X. (2023). Effect of process parameters on microstructure and tribological properties of Ni60A/Cr_3_C_2_ laser cladding on 60Si2Mn steel. Surf. Coat. Technol..

[85] Cao S., Liang J., Wang L., Zhou J. (2021). Effects of NiCr intermediate layer on microstructure and tribological property of laser cladding Cr_3_C_2_ reinforced Ni60A-Ag composite coating on copper alloy. Opt. Laser Technol..

[86] Yang C., Lu Y., Kong D. (2024). Laser cladded Ni625–xCr_3_C_2_ coatings: Microstructure, tribocorrosion and electrochemical properties. Surf. Coat. Technol..

[87] Mao K., Du Y., Cao H., Peng Y., He G., Liang Q., Tu J. (2024). Effects of Cr_3_C_2_ addition on microstructure and corrosion properties of Cr_3_C_2_/15–5PH composite coatings on 12Cr13 by laser cladding. Mater. Today Commun..

[88] Wang Y., Feng Y., Sun X., Liu S., Chen G. (2024). Effect of Process Parameters on the Microstructure and Wear Resistance of Fe3Al/Cr_3_C_2_ Composites. Coatings.

[89] Fan X., Li W., Yang J., Zhu S., Cui S., Li X., Shao L., Zhai H. (2023). Effect of Cr_3_C_2_ content on microstructure, mechanical and tribological properties of Ni3Al-based coatings. Surf. Coat. Technol..

[90] Aghili S.E., Shamanian M., Amini Najafabadi R., Keshavarzkermani A., Esmaeilizadeh R., Ali U., Marzbanrad E., Toyserkani E. (2020). Microstructure and oxidation behavior of NiCr-chromium carbides coating prepared by powder-fed laser cladding on titanium aluminide substrate. Ceram. Int..

[91] Aghili S.E., Shamanian M. (2019). Investigation of powder fed laser cladding of NiCr-chromium carbides single-tracks on titanium aluminide substrate. Opt. Laser Technol..

[92] Venkatesh L., Samajdar I., Tak M., Doherty R.D., Gundakaram R.C., Prasad K.S., Joshi S.V. (2015). Microstructure and phase evolution in laser clad chromium carbide-NiCrMoNb. Appl. Surf. Sci..

[93] Li Z., Yan H., Zhang P., Guo J., Yu Z., Ringsberg J.W. (2021). Improving surface resistance to wear and corrosion of nickel-aluminum bronze by laser-clad TaC/Co-based alloy composite coatings. Surf. Coat. Technol..

[94] Yu T., Tang H. (2022). Microstructure and high-temperature wear behavior of laser clad TaC-reinforced Ni-Al-Cr coating. Appl. Surf. Sci..

[95] Hu D., Liu Y., Chen H., Liu J., Wang M. (2021). Effect of TiC addition on the microstructure and properties of Ni3Ta–TaC reinforced Ni-based wear-resistant coating. Ceram. Int..

[96] Hu D., Liu Y., Chen H., Wang M., Liu J. (2021). Microstructure and properties of in-situ synthesized Ni3Ta-TaC reinforced Ni-based coatings by laser cladding. Surf. Coat. Technol..

[97] Liu Y., Ding T., Lv H., Hu D., Zhang Y., Chen H., Chen Y., She J. (2022). Microstructure and properties of Ta-reinforced cobalt based composite coatings processed by direct laser deposition. Surf. Coat. Technol..

